# Supplementary material for: Functional divergence and conservation in the QueC protein family (PF06508): from tRNA modification to anti-phage defense
Source: Biochem J. 2026 Jul 2;483(8):1357–67. doi: 10.1042/BCJ20260244 (PMC13329287; doi:10.1042/BCJ20260244)
Supplement: Supplementary Figure S1-S7, Tables S1-S4 and Raw Data S1-S3 [file BCJ-2026-0244_supp.pdf]

# Functional Divergence and Conservation in the QueC Protein Family (PF06508): From tRNA Modification to Anti-Phage Defense - Supplemental material

Kaitlynn Libby[1+], Crista Calia[2], Rahul Benda[1+], Valérie de Crécy-Lagard[1,3*], Geoffrey Hutinet[1,2*]

1 Department of Microbiology and Cell Science, University of Florida, Gainesville, FL 32611

2 Department of Biology, Haverford College, 370 Lancaster Ave, Haverford, PA 19041

3 University of Florida, Genetics Institute, Gainesville, Florida 32610

+ Present address :

KL: University of Florida, ICBR, Gainesville, FL 32610

RB: Evotec Biologics, Inc., Redmond, WA 98053

* corresponding authors: GH - ghutinet@haverford.edu, VdC - vcrecy@ufl.edu

**Supplemental Data 1. Proteins analyzed in the SSN.**

**Supplemental Data 2. Proteins in the neighborhood of the gene of interests.**

**Supplemental Data 3. ChimeraX file containing all protein structures.**

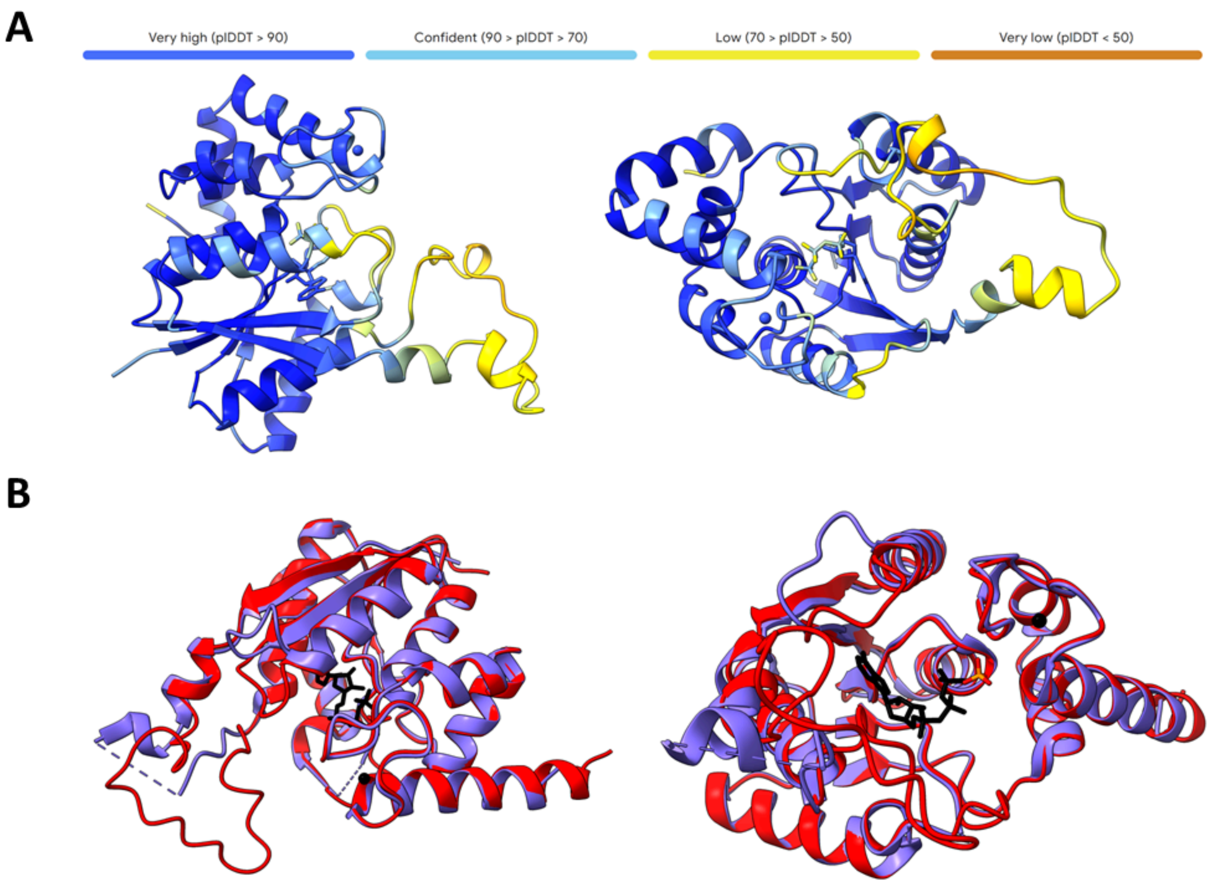


**Supplemental Figure 1. Predicted structure of *E. coli* MG1655 QueC.**

**(A)** E. coli MG1655 QueC structure was predicted using AlphaFold3 in presence of ATP and $Zn^{2+}$ and colored based on the pLDDT confidence score of the structure: very-low (orange), low (cyan), high (cyan), and very-high (blue). **(B)** The predicted structure in red was overlaid with the crystal structure of *B. subtilis* QueC (PDB: 3BL5, in purple). All structures are represented in different orientations.

A

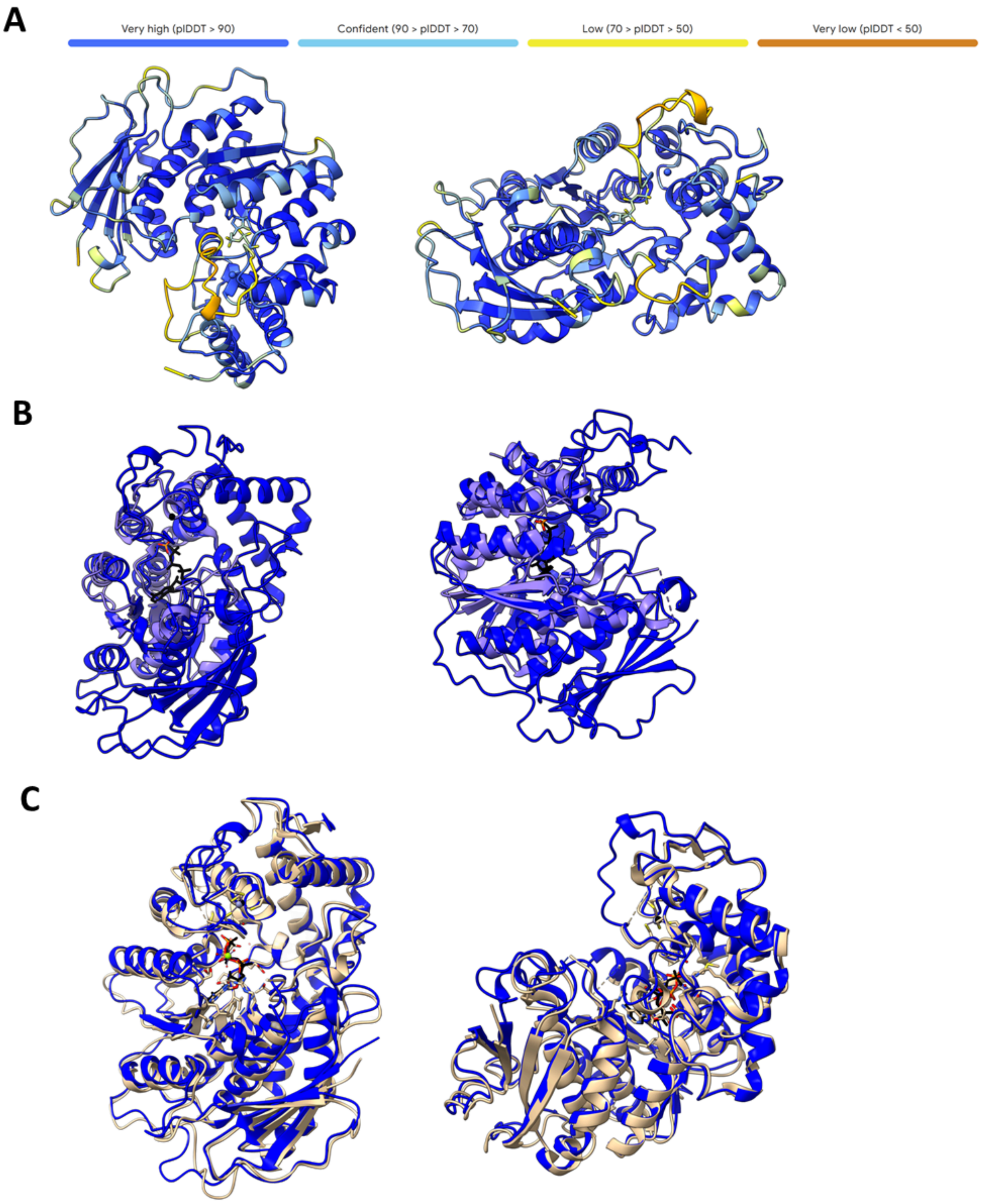


**Supplemental Figure 2. Predicted structure and conserved residues for Cluster 2.**

**(A)** QatC (sequence from  pLG027 [10]) structure was predicted using AlphaFold3 in presence of ATP and $Zn^{2+}$ and colored based on the pLDDT confidence score of the structure: very-low (orange), low (cyan), high (cyan), and very-high (blue).  **(B)** The predicted structure in dark blue was overlaid with the crystal structure of *B. subtilis* QueC (PDB: 3BL5, in purple). **(C)** The predicted structure in dark blue was overlaid with the crystal structure of *Pseudomonas aeruginosa* QatC (PDB: 9ZEE, in beige).

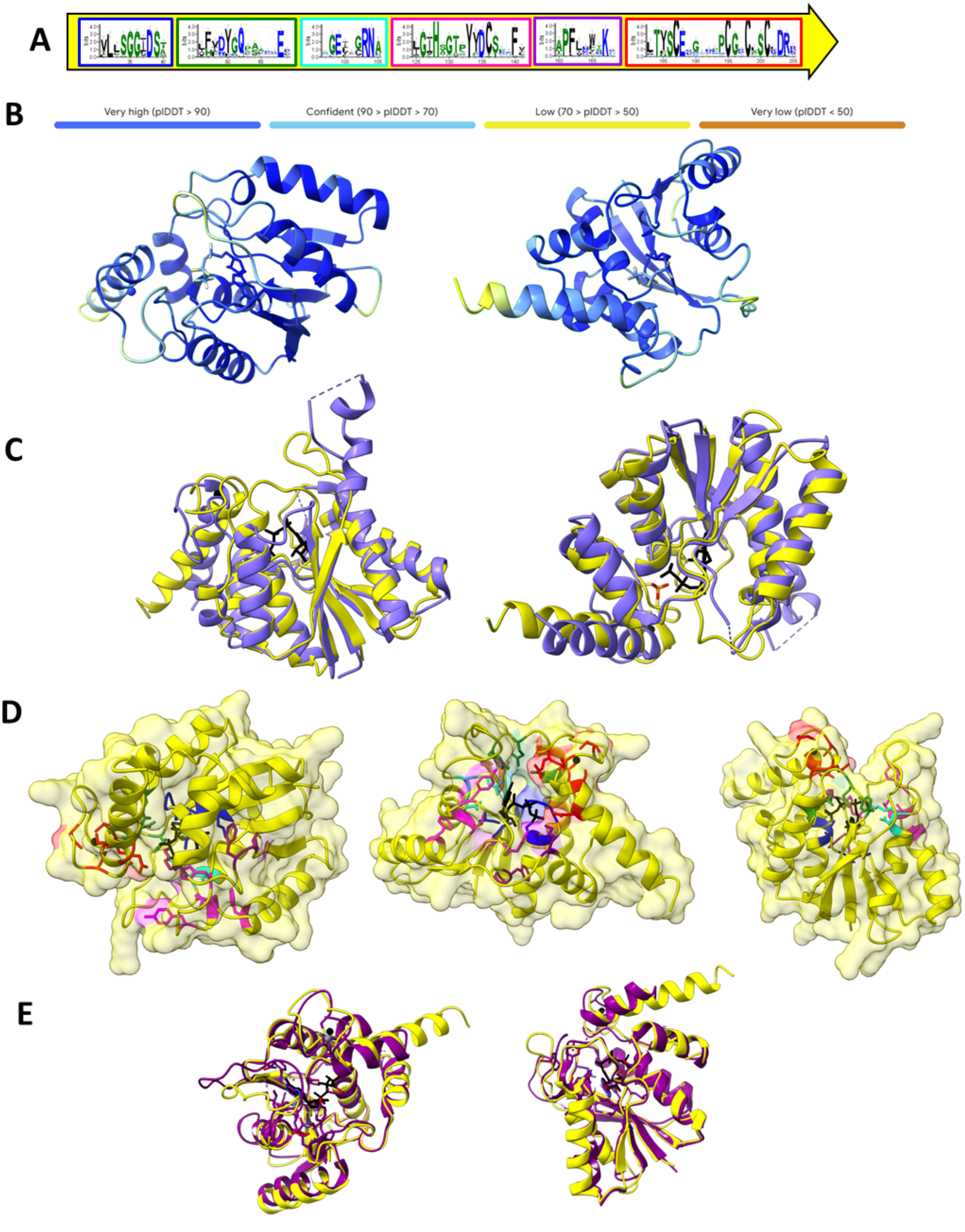
A
B
Very high (plDDT > 90)
Confident (90 > plDDT > 70)
Low (70 > plDDT > 50)
Very low (plDDT < 50)
C
D
E

**Supplemental Figure 3. Predicted structure and conserved residues for Cluster 9.**

**(A)** Cluster 9 sequence represented as a yellow arrow with conserved residues shown as conserved motifs. **(B)** Cluster 9 (*B. subtilis* sequence A0A5D4PAF6) structure was predicted using AlphaFold3 in presence of ATP and $Zn^{2+}$ and colored based on the pLDDT confidence score of the structure: very-low (orange), low (cyan), high (cyan), and very-high (blue). **(C)** The predicted structure in yellow was overlaid with the crystal structure of *B. subtilis* QueC (PDB: 3BL5, in purple). **(D)** Conserved residues were mapped onto the predicted structure, and colored based on each motif detected in panel A. All structures are represented in different orientations. **(E)** The predicted structure in dark blue was overlaid with the crystal structure of *Rhizobiales sp.* Cap9 (PDB: 9NTO, in purple).

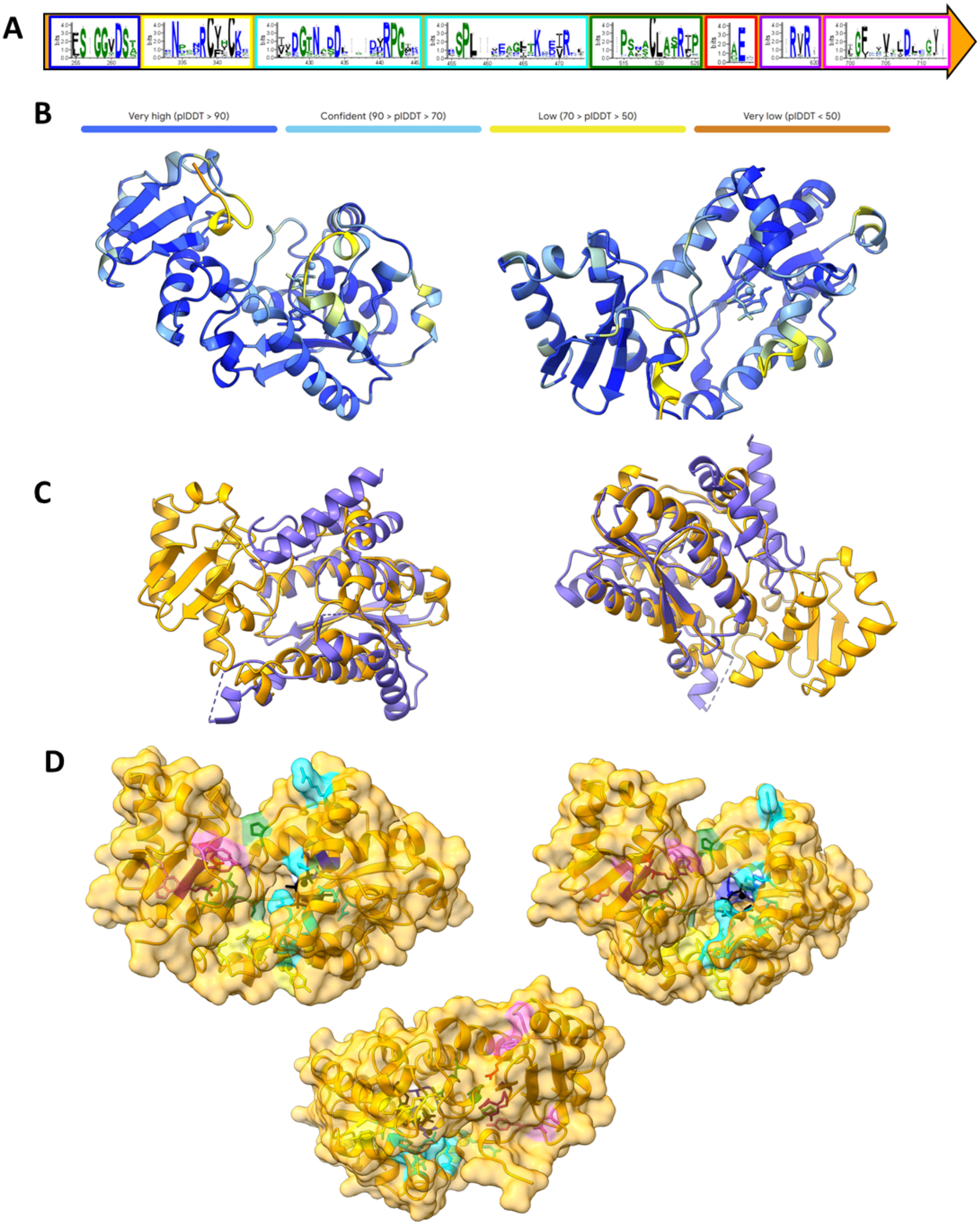
A
B
Very high (pIDDT > 90)
Confident (90 > pIDDT > 70)
Low (70 > pIDDT > 50)
Very low (pIDDT < 50)
C
D

**Supplemental Figure 4. Predicted structure and conserved residues for Cluster 3.**

**(A)** Cluster 3 sequence represented as an orange arrow with conserved residues shown as conserved motifs. **(B)** Cluster 3 (sequence from *Desulfotignum phosphitoxidans* DSM 13687, S0FY02) structure was predicted using AlphaFold3 in presence of ATP and $Zn^{2+}$ and colored based on the pLDDT confidence score of the structure: very-low (orange), low (cyan), high (cyan), and very-high (blue). **(C)** The predicted structure in orange was overlaid with the crystal structure of *B. subtilis* QueC (PDB: 3BL5, in purple). **(D)** Conserved residues were mapped onto the predicted structure, and colored based on each motif detected in panel A. All structures are represented in different orientations.

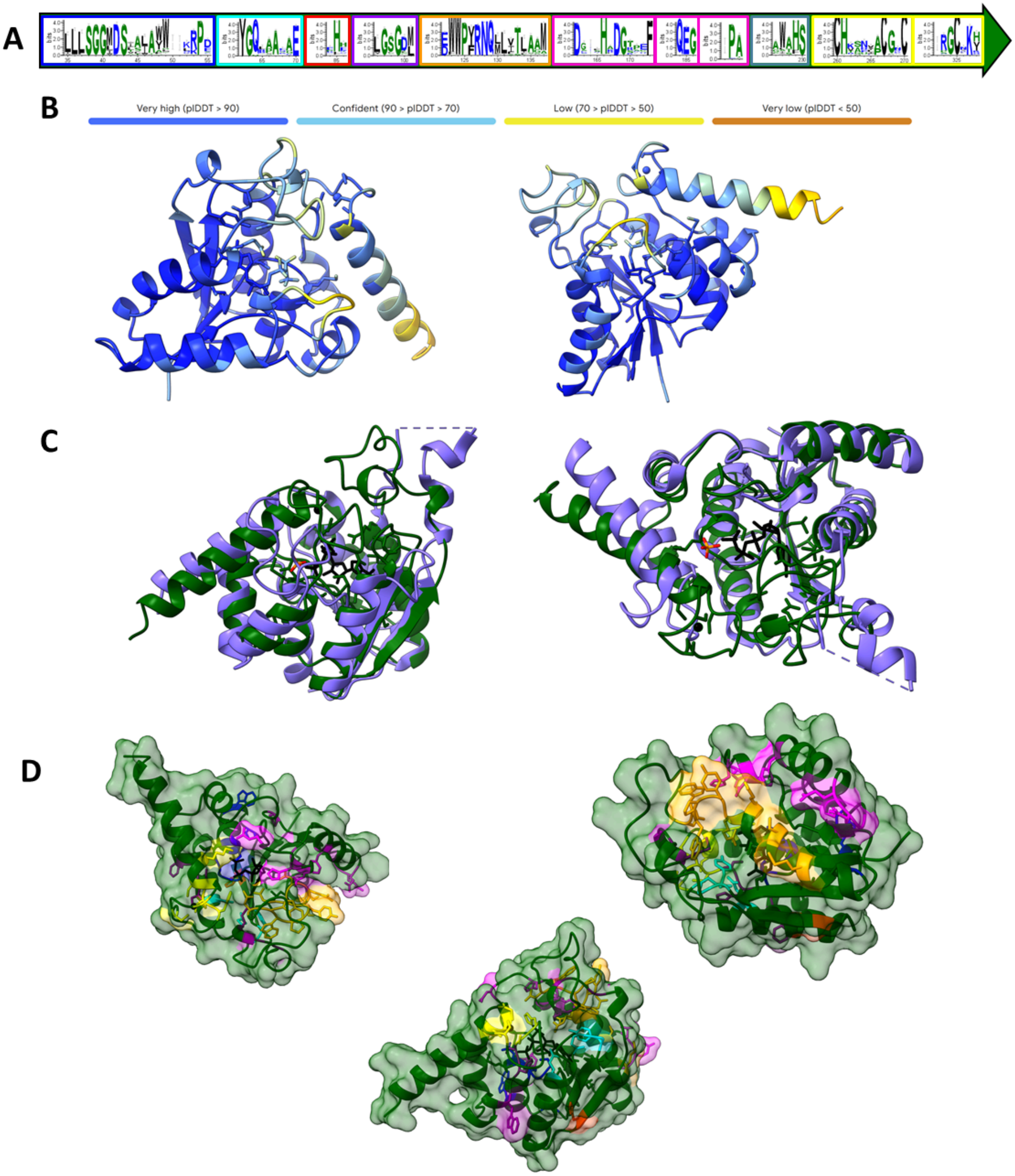


**Supplemental Figure 5. Predicted structure and conserved residues for Cluster 4.**

**(A)** Cluster 4 sequence represented as a green arrow with conserved residues shown as conserved motifs. **(B)** Cluster 4 (sequence from *Salmonella enterica subsp. enterica serovar Mikawa*, A0A5J2UZ21)

structure was predicted using AlphaFold3 in presence of ATP and $Zn^{2+}$ and colored based on the pLDDT confidence score of the structure: very-low (orange), low (cyan), high (cyan), and very-high (blue). **(C)** The predicted structure in green was overlaid with the crystal structure of *B. subtilis* QueC (PDB: 3BL5, in purple). **(D)** Conserved residues were mapped onto the predicted structure, and colored based on each motif detected in panel A. All structures are represented in different orientations.

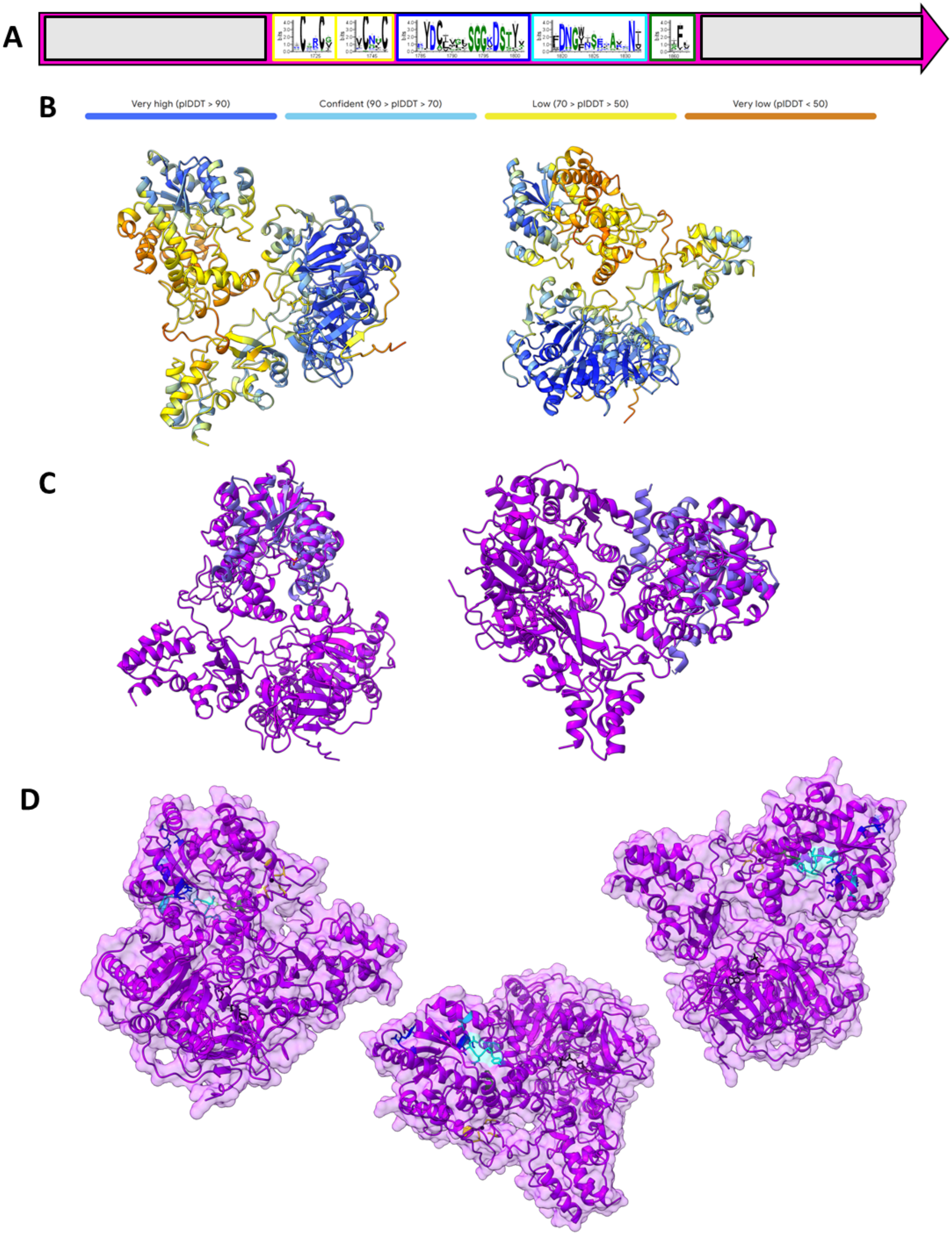
A
B
Very high (pIDDT > 90)
Confident (90 > pIDDT > 70)
Low (70 > pIDDT > 50)
Very low (pIDDT < 50)
C
D

**Supplemental Figure 6. Predicted structure and conserved residues for Cluster 5.**

**(A)** Cluster 5 sequence represented as a magenta arrow with conserved residues shown as conserved motifs. Grey boxes represent additional domains to the Rossman fold, and were not analyzed. **(B)** Cluster 5 (sequence from *Xanthomonas albilineans*, D2UDP3) structure was predicted using AlphaFold3 in presence of ATP and $Zn^{2+}$ and colored based on the pLDDT confidence score of the structure: very-low (orange), low (cyan), high (cyan), and very-high (blue). **(C)** The predicted structure in magenta was overlaid with the crystal structure of *B. subtilis* QueC (PDB: 3BL5, in purple). **(D)** Conserved residues were mapped onto the predicted structure, and colored based on each motif detected in panel A. All structures are represented in different orientations.

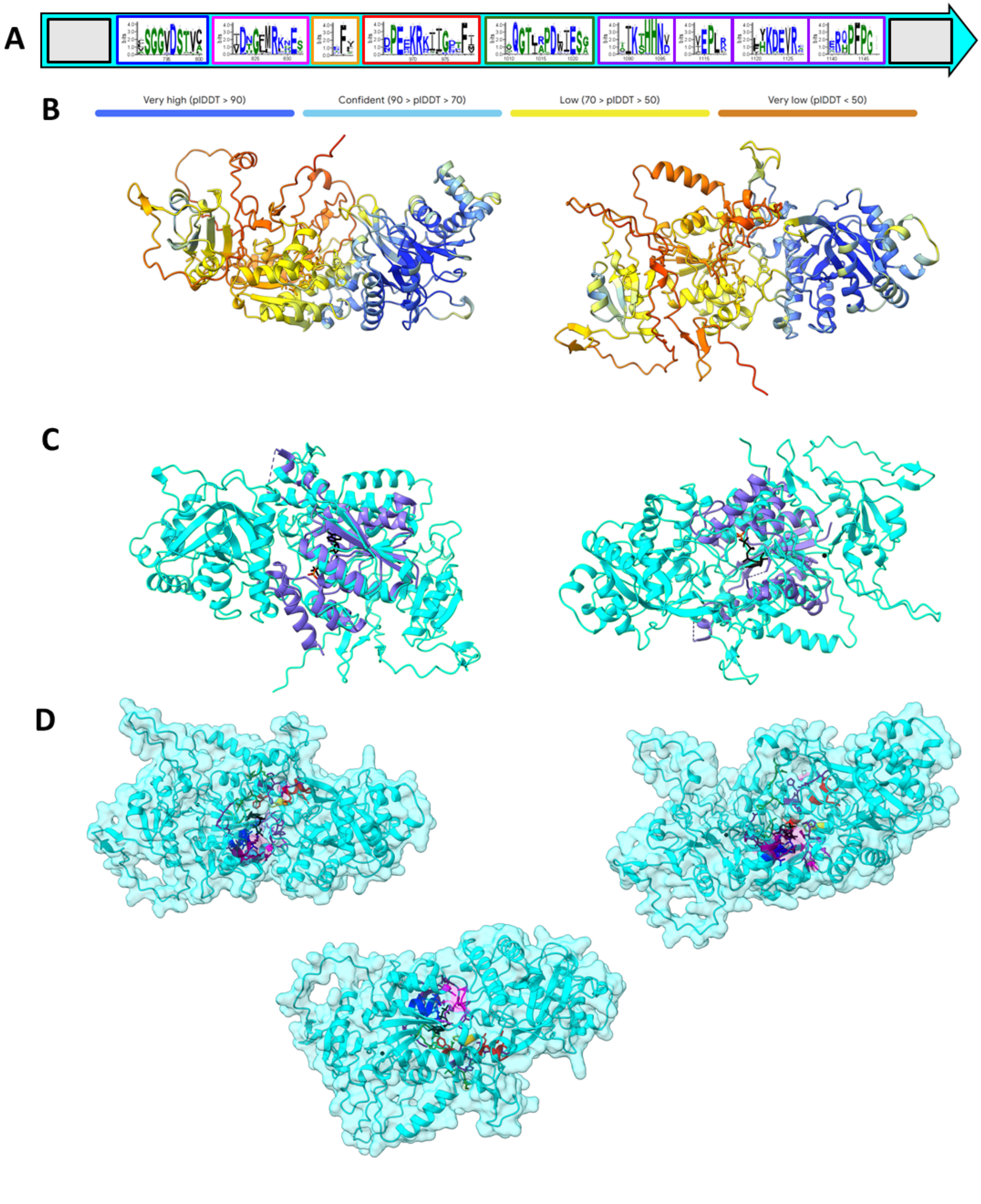


**Supplemental Figure 7. Predicted structure and conserved residues for Cluster 6.**

**(A)** Cluster 6 sequence represented as a cyan arrow with conserved residues shown as conserved motifs. Grey boxes represent additional domains to the Rossman fold, and were not analyzed. **(B)** Cluster 6 (sequence from *Heterocephalus glaber*, G5B263) structure was predicted using AlphaFold3 in presence of ATP and $Zn^{2+}$ and colored based on the pLDDT confidence score of the structure: very-low (orange), low (cyan), high (cyan), and very-high (blue). **(C)** The predicted structure in cyan was overlaid with the crystal structure of *B. subtilis* QueC (PDB: 3BL5, in purple). **(D)** Conserved residues were mapped onto the predicted structure, and colored based on each motif detected in panel A. All structures are represented in different orientations.

**Supplemental Table 1. Analyzed Eukaryotes in Cluster 1**

| node id | Organism | Gene Name | top result that isn't itself (if applicable) |
|---|---|---|---|
| A0A0J7NJF6 | Lasius niger | RF55_7356 | Acetobacteraceae bacterium (89.4%) |
| A0A1I7YRA3 | Steinernema glaseri. | None | Alcaligenes sp. EGD-AK7 (95.3%) |
| A0A1L5YBT0 | Paulinella micropora. | PMNZ_384 | Synechococcus sp. RS9916 (67.9%) |
| A0A1L5YBT0 | Paulinella micropora. | PMNZ_384 | |
| A0A2H4ZPB6 | Paulinella longichromatophora. | PLO_380 | |
| A0A518KB09 | Symbiodinium sp. CCMP2456. | Spa11_31780 | Botrimarina colliarenosi (59.3%) |
| A0A5E4M7N2 | Cinara cedri. | CINCED_3A021500 | Serratia proteamaculans (strain 568) (75%) |
| A0A7J7AE15 | Abscondita terminalis. | FQR65_LT20447 | Acinetobacter guillouiae NIPH 991 (97.2%) |
| A0A7J7AJS3 | Abscondita terminalis. | FQR65_LT20048 | Pelomonas puraquae (94.8%) |
| A0A7R8WUF2 | Cyprideis torosa. | CTOB1V02_LOCUS15247 | Paramesorhizobium deserti (45.6%) |
| A0A7R8WW36 | Cyprideis torosa. | CTOB1V02_LOCUS13378 | Neptunomonas concharum (89.6%) |
| A0A7S0Q9F8 | Coccolithus braarudii. | CPEL01642_LOCUS20965 | Lichenicola cladoniae (69.9%) |
| A0A7S3BKH0 | Haptolina ericina. | HERI1096_LOCUS31437 | Physcomitrium patens (Spreading-leaved earth moss) (Physcomitrella patens) (39.9%) |
| A0A7S3VVP5 | Strombidinopsis acuminata. | SACU0126_LOCUS1315 | Caulobacteraceae bacterium (62.9%) |
| A0A7V5M2J0 | bacterium. | ENL98_11005 | |
| A0A812IS58 | Symbiodinium microadriaticum | SMIC04503_LOCUS45 | Marinobacterium sp. 3-1745 (69.1%) |
| A0A812QUJ7 | Symbiodinium microadriaticum | SMIC04503_LOCUS11741 | Hyphomonas sp. CY54-11-8 (71.7%) |
| A0A812RWX3 | Symbiodinium pilosum | SPIL2461_LOCUS11338 | Cowellia chukchiensis (45.9%) |
| A0A813CDS7 | Symbiodinium necroappetens. | SNEC2469_LOCUS34832 | Symbiodinium sp. CCMP2592 (41.5%) |
| A0A817A071 | Rotaria magnacalcarata. | None | Rickettsia akari (strain Hartford) (62.1%) |

| | | | |
|---|---|---|---|
| A0A834MB60 | Rhynchophorus ferrugineus | GWI33_012054 | Acintebacter marinus (67%) |
| A0A8J6C679 | Diacronema lutheri | KFE25_001342 | Opitutus sp. ER46 (77.8%) |
| F7XV14 | Midichloria mitochondrii (strain IricVA) | midi_00194 | |
| T1HJC6 | Rhodnius prolixus | None | Brenneria rubrifaciens (85%) |

**Supplemental Table 2. Quantification of protein encoded in the neighborhood of the genes of interest for each cluster.**

| cluster | pFAM | #pFAM_neighbors | #pFAM_in_operon | Annotation | %of_genome | %in_operon |
|---|---|---|---|---|---|---|
| 1 | PF01242 | 7683 | 7285 | PTPS | 32.61450949 | 30.92499045 |
| 1 | PF04055 | 6160 | 5102 | Radical_SAM | 26.1493399 | 21.65810587 |
| 1 | PF07690 | 5946 | 443 | MFS_1 | 25.24090504 | 1.880545061 |
| 1 | PF00005 | 5499 | 587 | ABC_tran | 23.34337989 | 2.491828331 |
| 1 | PF04055-PF13353 | 5100 | 4373 | Radical_SAM;Fer4_12 | 21.64961583 | 18.56348431 |
| 2 | PF07693 | 202 | 199 | KAP_NTPase | 34.29541596 | 33.7860781 |
| 2 | PF01026 | 198 | 191 | TatD_DNase | 33.61629881 | 32.4278438 |
| 3 | PF01969 | 292 | 278 | Ni_insertion | 54.37616387 | 51.76908752 |
| 3 | PF00731 | 252 | 154 | AIRC | 46.9273743 | 28.67783985 |
| 3 | PF00005 | 185 | 68 | ABC_tran | 34.45065177 | 12.66294227 |
| 3 | PF00266 | 135 | 112 | Aminotran_5 | 25.1396648 | 20.8566108 |
| 3 | PF00528 | 112 | 54 | BPD_transp_1 | 20.8566108 | 10.05586592 |

| | | | | | | |
|---|---|---|---|---|---|---|
| 4 | PF00294-PF05014 | 237 | 234 | PfkB;Nuc_deoxyrib_tr | 89.09774436 | 87.96992481 |
| 4 | PF01381 | 83 | 9 | HTH_3 | 31.20300752 | 3.383458647 |
| 4 | PF00881 | 60 | 53 | Nitroreductase | 22.55639098 | 19.92481203 |
| 4 | PF00126-PF03466 | 55 | 2 | HTH_1;LysR_substrate | 20.67669173 | 0.7518796992 |
| 5 | PF01041 | 82 | 62 | DegT_DnrJ_EryC1 | 51.25 | 38.75 |
| 5 | PF00534-PF13439 | 64 | 49 | Glycos_transf_1;Glyco_transf_4 | 40 | 30.625 |
| 5 | PF00535 | 61 | 44 | Glycos_transf_2 | 38.125 | 27.5 |
| 5 | PF00977 | 57 | 54 | His_biosynth | 35.625 | 33.75 |
| 5 | PF00534 | 53 | 50 | Glycos_transf_1 | 33.125 | 31.25 |
| 5 | PF00117 | 53 | 49 | GATase | 33.125 | 30.625 |
| 5 | PF01370 | 52 | 34 | Epimerase | 32.5 | 21.25 |
| 5 | PF00550 | 48 | 44 | PP-binding | 30 | 27.5 |
| 5 | PF00733-PF13537 | 38 | 35 | Asn_synthase;GATase_7 | 23.75 | 21.875 |
| 5 | PF00501-PF13193 | 36 | 30 | AMP-binding;AMP-binding_C | 22.5 | 18.75 |
| 5 | PF01408-PF02894 | 36 | 25 | GFO_IDH_MocA;GFO_IDH_MocA_C | 22.5 | 15.625 |
| 5 | PF02350 | 35 | 30 | Epimerase_2 | 21.875 | 18.75 |
| 6 | PF00731 | 32 | 32 | AIRC | 34.40860215 | 34.40860215 |
| 6 | PF00117 | 24 | 20 | GATase | 25.80645161 | 21.50537634 |

| | | | | | | |
|---|---|---|---|---|---|---|
| 7 | PF00528 | 137 | 61 | BPD_transp_1 | 165.060241 | 73.4939759 |
| 7 | PF00005-PF08352 | 86 | 0 | ABC_tran;oligo_HPY | 103.6144578 | 0 |
| 7 | PF04055 | 73 | 59 | Radical_SAM | 87.95180723 | 71.08433735 |
| 7 | PF02355 | 68 | 0 | SecD_SecF | 81.92771084 | 0 |
| 7 | PF00892 | 62 | 0 | EamA | 74.69879518 | 0 |
| 7 | PF01411-PF02272-PF07973 | 55 | 0 | tRNA-synt_2c;DHHA1;tRNA_SAD | 66.26506024 | 0 |
| 7 | PF01813 | 51 | 0 | ATP-synt_D | 61.44578313 | 0 |
| 7 | PF00137 | 50 | 1 | ATP-synt_C | 60.24096386 | 1.204819277 |
| 7 | PF01991 | 49 | 0 | vATP-synt_E | 59.03614458 | 0 |
| 7 | PF01992 | 49 | 0 | vATP-synt_AC39 | 59.03614458 | 0 |
| 7 | PF00006-PF02874 | 49 | 0 | ATP-synt_ab;ATP-synt_ab_N | 59.03614458 | 0 |
| 7 | PF01990 | 49 | 0 | ATP-synt_F | 59.03614458 | 0 |
| 7 | PF01979 | 48 | 39 | Amidohydro_1 | 57.8313253 | 46.98795181 |
| 7 | PF01496 | 48 | 0 | V_ATPase_I | 57.8313253 | 0 |
| 7 | PF06745 | 46 | 0 | ATPase | 55.42168675 | 0 |
| 7 | PF02080-PF02254 | 43 | 0 | TrkA_C;TrkA_N | 51.80722892 | 0 |
| 7 | PF00496 | 43 | 0 | SBP_bac_5 | 51.80722892 | 0 |
| 7 | PF00006-PF02874-PF16886 | 40 | 0 | ATP-synt_ab;ATP-synt_ab_N;ATP-synt_ab_Xtn | 48.19277108 | 0 |

| | | | | | | |
|---|---|---|---|---|---|---|
| 7 | PF00175-PF10418 | 37 | 36 | NAD_binding_1;DHODB_Fe-S_bind | 44.57831325 | 43.37349398 |
| 7 | PF02675 | 35 | 0 | AdoMet_dc | 42.1686747 | 0 |
| 7 | PF02540 | 32 | 0 | NAD_synthase | 38.55421687 | 0 |
| 7 | PF01886 | 31 | 0 | DUF61 | 37.34939759 | 0 |
| 7 | PF00120-PF03951 | 31 | 0 | Gln-synt_C;Gln-synt_N | 37.34939759 | 0 |
| 7 | PF00528-PF12911 | 29 | 0 | BPD_transp_1;OppC_N | 34.93975904 | 0 |
| 7 | PF01896 | 28 | 0 | DNA_primase_S | 33.73493976 | 0 |
| 7 | PF04104 | 22 | 0 | DNA_primase_lrg | 26.5060241 | 0 |
| 7 | PF00015 | 22 | 0 | MCPsignal | 26.5060241 | 0 |
| 7 | PF07690 | 21 | 0 | MFS_1 | 25.30120482 | 0 |
| 7 | PF19583 | 21 | 0 | ODP | 25.30120482 | 0 |
| 7 | PF02355-PF07549 | 17 | 0 | SecD_SecF;Sec_GG | 20.48192771 | 0 |
| 7 | PF03657 | 17 | 0 | UPF0113 | 20.48192771 | 0 |
| 8 | PF01944 | 37 | 1 | SpoIIM | 50.68493151 | 1.369863014 |
| 8 | PF00579 | 36 | 1 | tRNA-synt_1b | 49.31506849 | 1.369863014 |
| 8 | PF00206-PF14698 | 34 | 5 | Lyase_1;ASL_C2 | 46.57534247 | 6.849315068 |
| 8 | PF00808 | 31 | 4 | CBFD_NFYB_HMF | 42.46575342 | 5.479452055 |
| 8 | PF01599 | 29 | 5 | Ribosomal_S27 | 39.7260274 | 6.849315068 |
| 8 | PF04019 | 29 | 5 | DUF359 | 39.7260274 | 6.849315068 |

| | | | | | | |
|---|---|---|---|---|---|---|
| 8 | PF01282 | 29 | 5 | Ribosomal_S24e | 39.7260274 | 6.849315068 |
| 8 | PF13197 | 28 | 2 | DUF4013 | 38.35616438 | 2.739726027 |
| 8 | PF00575-PF03876 | 26 | 3 | S1;SHS2_Rpb7-N | 35.61643836 | 4.109589041 |
| 8 | PF06093 | 26 | 3 | Spt4 | 35.61643836 | 4.109589041 |
| 8 | PF18477 | 25 | 3 | PIN_9 | 34.24657534 | 4.109589041 |
| 8 | PF01092 | 25 | 3 | Ribosomal_S6e | 34.24657534 | 4.109589041 |
| 8 | PF00155 | 23 | 0 | Aminotran_1_2 | 31.50684932 | 0 |
| 8 | PF01248 | 22 | 1 | Ribosomal_L7Ae | 30.1369863 | 1.369863014 |
| 8 | PF01246 | 22 | 1 | Ribosomal_L24e | 30.1369863 | 1.369863014 |
| 8 | PF01890-PF11760 | 22 | 0 | CbiG_C;CbiG_N | 30.1369863 | 0 |
| 8 | PF01200 | 22 | 1 | Ribosomal_S28e | 30.1369863 | 1.369863014 |
| 8 | PF04608 | 21 | 0 | PgpA | 28.76712329 | 0 |
| 8 | PF01472-PF17884 | 21 | 0 | PUA;DUF5591 | 28.76712329 | 0 |
| 8 | PF06325 | 21 | 0 | PrmA | 28.76712329 | 0 |
| 8 | PF00117-PF00988 | 21 | 20 | GATase;CPSase_sm_chain | 28.76712329 | 27.39726027 |
| 8 | PF00389-PF01842-PF02826-PF19304 | 21 | 0 | 2-Hacid_dh;ACT;2-Hacid_dh_C;PGDH_inter | 28.76712329 | 0 |
| 8 | PF00491 | 21 | 0 | Arginase | 28.76712329 | 0 |
| 8 | PF00892 | 21 | 0 | EamA | 28.76712329 | 0 |

| | | | | | | |
|---|---|---|---|---|---|---|
| 8 | PF04467 | 21 | 0 | DUF483 | 28.76712329 | 0 |
| 8 | PF02441-PF04127 | 21 | 0 | Flavoprotein;DFP | 28.76712329 | 0 |
| 8 | PF01837 | 21 | 0 | HcyBio | 28.76712329 | 0 |
| 8 | PF01564-PF17284 | 21 | 0 | Spermine_synth;Spermine_synt_N | 28.76712329 | 0 |
| 8 | PF02080-PF02254 | 21 | 0 | TrkA_C;TrkA_N | 28.76712329 | 0 |
| 8 | PF00334 | 21 | 1 | NDK | 28.76712329 | 1.369863014 |
| 8 | PF08443 | 21 | 0 | RimK | 28.76712329 | 0 |
| 8 | PF13086-PF13087 | 20 | 0 | AAA_11;AAA_12 | 27.39726027 | 0 |
| 8 | PF13500 | 20 | 0 | AAA_26 | 27.39726027 | 0 |
| 8 | PF01026 | 20 | 0 | TatD_DNase | 27.39726027 | 0 |
| 8 | PF03744 | 20 | 0 | BioW | 27.39726027 | 0 |
| 8 | PF01862 | 20 | 0 | PvlArgDC | 27.39726027 | 0 |
| 8 | PF01467 | 20 | 0 | CTP_transf_like | 27.39726027 | 0 |
| 8 | PF00312-PF08069 | 20 | 0 | Ribosomal_S15;Ribosomal_S13_N | 27.39726027 | 0 |
| 8 | PF01913-PF02741 | 20 | 0 | FTR;FTR_C | 27.39726027 | 0 |
| 8 | PF02915 | 20 | 0 | Rubrerythrin | 27.39726027 | 0 |
| 8 | PF08489 | 20 | 0 | DUF1743 | 27.39726027 | 0 |
| 8 | PF00224-PF02887 | 20 | 0 | PK;PK_C | 27.39726027 | 0 |

| | | | | | | |
|---|---|---|---|---|---|---|
| 8 | PF01949 | 20 | 0 | DUF99 | 27.39726027 | 0 |
| 8 | PF02675 | 20 | 0 | AdoMet_dc | 27.39726027 | 0 |
| 8 | PF00291 | 19 | 1 | PALP | 26.02739726 | 1.369863014 |
| 8 | PF00009-PF11987-PF14578 | 18 | 1 | GTP_EFTU;IF-2;GTP_EFTU_D4 | 24.65753425 | 1.369863014 |
| 8 | PF00809-PF14251 | 17 | 0 | Pterin_bind;DUF4346 | 23.28767123 | 0 |
| 8 | PF00132 | 15 | 0 | Hexapep | 20.54794521 | 0 |
| 8 | PF01161 | 15 | 7 | PBP | 20.54794521 | 9.589041096 |
| 9 | PF18144 | 37 | 36 | SMODS | 67.27272727 | 65.45454545 |
| 9 | PF01420 | 13 | 8 | Methylase_S | 23.63636364 | 14.54545455 |
| 9 | PF00589 | 12 | 2 | Phage_integrase | 21.81818182 | 3.636363636 |
| 10 | PF00586-PF02769 | 39 | 38 | AIRS;AIRS_C | 76.47058824 | 74.50980392 |
| 10 | PF02421-PF07664-PF07670-PF17910 | 39 | 0 | FeoB_N;FeoB_C;Gate;FeoB_Cyto | 76.47058824 | 0 |
| 10 | PF03463-PF03464-PF03465 | 39 | 0 | eRF1_1;eRF1_2;eRF1_3 | 76.47058824 | 0 |
| 10 | PF01523-PF19289-PF19290 | 38 | 0 | PmbA_TldD_1st;PmbA_TldD_3rd;PmbA_TldD_2nd | 74.50980392 | 0 |
| 10 | PF04023 | 38 | 0 | FeoA | 74.50980392 | 0 |
| 10 | PF00557-PF01321 | 36 | 0 | Peptidase_M24;Creatinase_N | 70.58823529 | 0 |

| | | | | | | |
|---|---|---|---|---|---|---|
| 10 | PF09685 | 36 | 36 | DUF4870 | 70.58823529 | 70.58823529 |
| 10 | PF00483 | 36 | 0 | NTP_transferase | 70.58823529 | 0 |
| 10 | PF02005 | 33 | 0 | TRM | 64.70588235 | 0 |
| 10 | PF01247 | 33 | 0 | Ribosomal_L35Ae | 64.70588235 | 0 |
| 10 | PF13412 | 30 | 0 | HTH_24 | 58.82352941 | 0 |
| 10 | PF01988-PF02915 | 29 | 0 | VIT1;Rubrerythrin | 56.8627451 | 0 |
| 10 | PF00587-PF03129 | 29 | 0 | tRNA-synt_2b;HGTP_anticodon | 56.8627451 | 0 |
| 10 | PF01554 | 28 | 0 | MatE | 54.90196078 | 0 |
| 10 | PF01928 | 28 | 0 | CYTH | 54.90196078 | 0 |
| 10 | PF02441 | 28 | 0 | Flavoprotein | 54.90196078 | 0 |
| 10 | PF02457-PF21756 | 28 | 0 | DAC;DacZ_T | 54.90196078 | 0 |
| 10 | PF01423 | 27 | 0 | LSM | 52.94117647 | 0 |
| 10 | PF00702 | 27 | 0 | Hydrolase | 52.94117647 | 0 |
| 10 | PF01907 | 27 | 0 | Ribosomal_L37e | 52.94117647 | 0 |
| 10 | PF01926-PF02824-PF08438 | 25 | 0 | MMR_HSR1;TGS;YGR210-like_G4 | 49.01960784 | 0 |
| 10 | PF02594 | 25 | 0 | DUF167 | 49.01960784 | 0 |
| 10 | PF04167 | 24 | 0 | DUF402 | 47.05882353 | 0 |
| 10 | PF01177 | 21 | 0 | Asp_Glu_race | 41.17647059 | 0 |
| 10 | PF00005 | 21 | 0 | ABC_tran | 41.17647059 | 0 |

| | | | | | | |
|---|---|---|---|---|---|---|
| 10 | PF07883 | 21 | 0 | Cupin_2 | 41.17647059 | 0 |
| 10 | PF01113-PF02800 | 21 | 0 | DapB_N;Gp_dh_C | 41.17647059 | 0 |
| 10 | PF01909 | 21 | 11 | NTP_transf_2 | 41.17647059 | 21.56862745 |
| 10 | PF00994 | 19 | 0 | MoCF_biosynth | 37.25490196 | 0 |
| 10 | PF01850 | 17 | 0 | PIN | 33.33333333 | 0 |
| 10 | PF01544 | 16 | 0 | CorA | 31.37254902 | 0 |
| 10 | PF00528 | 16 | 0 | BPD_transp_1 | 31.37254902 | 0 |
| 10 | PF01008 | 14 | 0 | IF-2B | 27.45098039 | 0 |
| 10 | PF02475-PF18093 | 13 | 0 | Met_10;Trm5_N | 25.49019608 | 0 |
| 10 | PF04021 | 12 | 0 | Class_IIIsignal | 23.52941176 | 0 |
| 10 | PF00753 | 11 | 0 | Lactamase_B | 21.56862745 | 0 |

**Supplemental Table 3. Primer list.**

| Primer name | Primer Sequence | Description |
|---|---|---|
| GO444 | GCACGCCATGGAACGTGCTGTCGTTG | NcoI + start of QueC Ec |
| GO445 | GCACGCCTGCAGGTTACCTCAACCCGGTTTTCTG | SbfI + end of QueC Ec |
| GO451 | TCCCGGATTGCCGCG | QueC Ec mutagenesis FW, A392>T => **Y131F** |
| GO452 | AGCCGGAGAAATCC | QueC Ec mutagenesis RV, A392>T => Y131F |
| GO453 | GCCGGTCAGCGGCATCG | QueC Ec mutagenesis FW, T103A104>GC => **Y35A** |
| GO454 | ATCGAACGTCACGCAATGG | QueC Ec mutagenesis RV, T103A104>GC => Y35A |
| GO455 | GCGCGGCATCGCGCAG | QueC Ec mutagenesis FW, C109A110>GC => **Q37A** |
| GO456 | ACCGTAATCGAACGTCACG | QueC Ec mutagenesis RV, C109A110>GC => Q37A |
| GO457 | CGTCGCGCAGAAATCGACG | QueC Ec mutagenesis FW, C115A116>GC => **H39A** |
| GO458 | CCGCTGACCGTAATCGAAC | QueC Ec mutagenesis RV, C115A116>GC => H39A |
| GO459 | CAATCGACGTGGCACGC | QueC Ec mutagenesis FW, A125>C => **E42A** |
| GO460 | CTGCGCGATGCCGC | QueC Ec mutagenesis RV, A125>C => E42A |
| GO461 | GCTAATATTTTGTTCCTGACGCTGG | QueC Ec mutagenesis FW, C295G296>GC => **R99A** |
| GO462 | CCCTGGGACAAACGTATTC | QueC Ec mutagenesis RV, C295G296>GC => R99A |
| GO463 | GCTATTTTGTTCCTGACGCTGG | QueC Ec mutagenesis FW, A298A299>GC => **N100A** |
| GO464 | ACGCCCTGGGACAAAC | QueC Ec mutagenesis RV, A298A299>GC => N100A |
| GO465 | GCCCCGGATTGCCGCG | QueC Ec mutagenesis FW, T391A392>GC => **Y131A** |
| GO466 | CGAAACGGATTTCTCCGGC | QueC Ec mutagenesis RV, T391A392>GC => Y131A |

| | | |
|---|---|---|
| GO467 | GCCCGCGATGAGTTTGTGAAAG | QueC Ec mutagenesis FW, T400G401>GC => **C134A** |
| GO468 | ATCCGGGTAGCCGGAG | QueC Ec mutagenesis RV, T400G401>GC => C134A |
| GO469 | GCAGCGGAAACCTGGGC | QueC Ec mutagenesis FW, A493A494>GC => **K165A** |
| GO470 | ATCAATCCACATCAGCGGC | QueC Ec mutagenesis RV, A493A494>GC => K165A |
| GO471 | GCTAACGGCTTTAAAGGCGAC | QueC Ec mutagenesis FW, T565A566>GC => **Y189A** |
| GO472 | GCAGGTCAACGTTTCGTTAC | QueC Ec mutagenesis RV, T565A566>GC => Y189A |
| GO473 | GCCGCCAACGGTTTGAATCATTATC | QueC Ec mutagenesis FW, C696G697>GC => **R206A** |
| GO474 | TAAATTACATGCCGCACAATGAC | QueC Ec mutagenesis RV, C696G697>GC => R206A |
| GO573 | ATGATCCATGGGCCATCATACCTTAGTTG | NcoI + start of Ec QatC |
| GO574 | ATGATCCTGCAGGTCATGTAAGGGGCCTCG | SbfI + end of Ec QatC |

**Supplemental Table 4. Strain list.**

| Strain name | *Species* | background | Plasmid | Origin |
|---|---|---|---|---|
| GJH1132 | *Escherishia coli* | MG1655 | pBAD24 | [2] |
| GJH1283 | *Escherishia coli* | MG1655 queC::kanR | pBAD24 | [2] |
| GJH2331 | *Escherishia coli* | MG1655 queC::kanR | pBAD24-Ec QueC | [2] |
| GJH2339 | *Escherishia coli* | MG1655 queC::kanR | pBAD24-Ec QueC Y131F | This study |
| GJH2341 | *Escherishia coli* | MG1655 queC::kanR | pBAD24-Ec QueC Y35A | This study |
| GJH2343 | *Escherishia coli* | MG1655 queC::kanR | pBAD24-Ec QueC Q37A | This study |
| GJH2345 | *Escherishia coli* | MG1655 queC::kanR | pBAD24-Ec QueC H39A | This study |
| GJH2347 | *Escherishia coli* | MG1655 queC::kanR | pBAD24-Ec QueC E42A | This study |
| GJH2349 | *Escherishia coli* | MG1655 queC::kanR | pBAD24-Ec QueC R99A | This study |
| GJH2351 | *Escherishia coli* | MG1655 queC::kanR | pBAD24-Ec QueC N100A | This study |
| GJH2353 | *Escherishia coli* | MG1655 queC::kanR | pBAD24-Ec QueC Y131A | This study |
| GJH2355 | *Escherishia coli* | MG1655 queC::kanR | pBAD24-Ec QueC C134A | This study |
| GJH2357 | *Escherishia coli* | MG1655 queC::kanR | pBAD24-Ec QueC K165A | This study |
| GJH2359 | *Escherishia coli* | MG1655 queC::kanR | pBAD24-Ec QueC Y189A | This study |
| GJH2361 | *Escherishia coli* | MG1655 queC::kanR | pBAD24-Ec QueC R206A | This study |
| GJH2642 | *Escherishia coli* | MG1655 | pLG027 | [10] |
| GJH2647 | *Escherishia coli* | MG1655 queC::kanR | pBAD24-qatC Ec | This study |
| GJH2702 | *Escherishia coli* | MG1655 | pACYC184 | [10] |

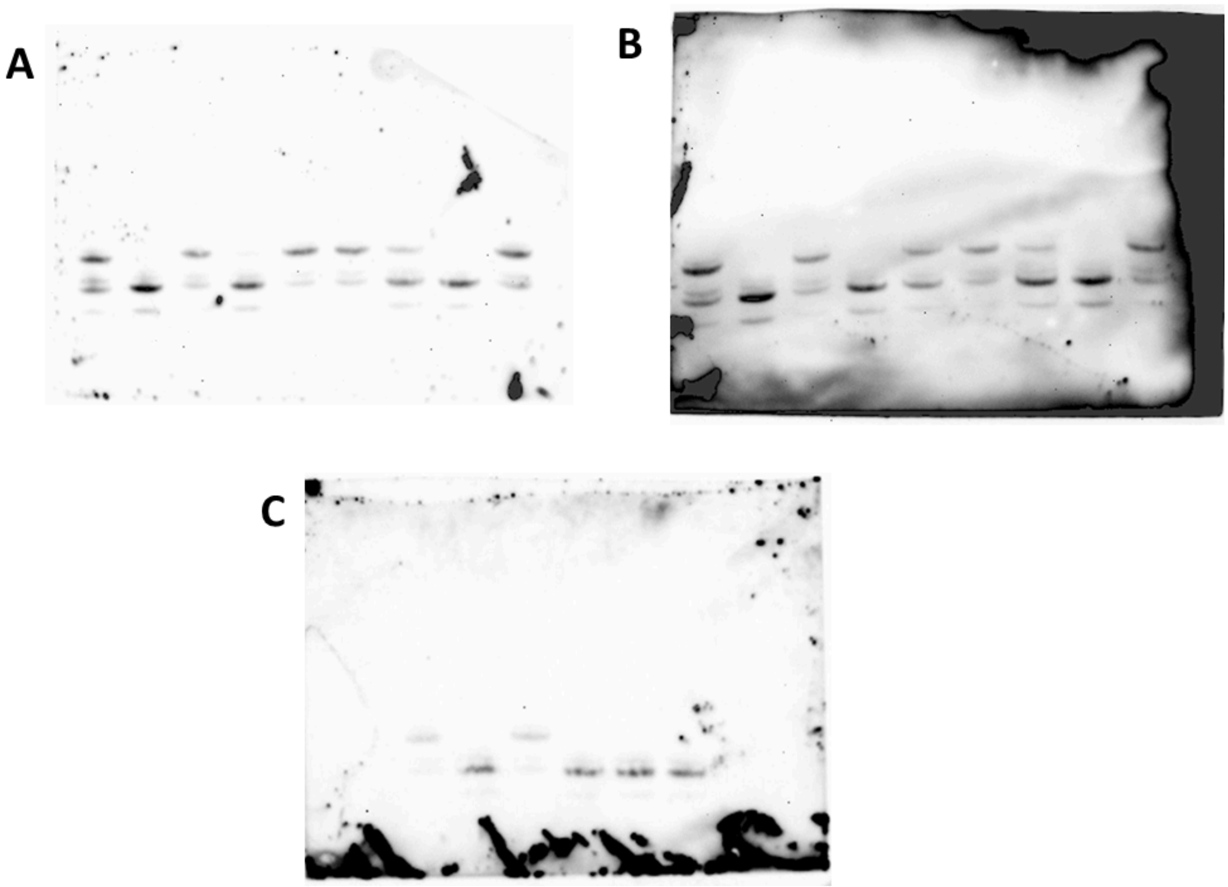


**Raw Data 1. Q-detection northern blots.**

(**A** and **B**) Uncropped northern blots represented in Figure 2D. (**C**) Uncropped northern blots represented in Figure 4C. The two additional lanes are biological replicates of the QatC complementation of QueC.

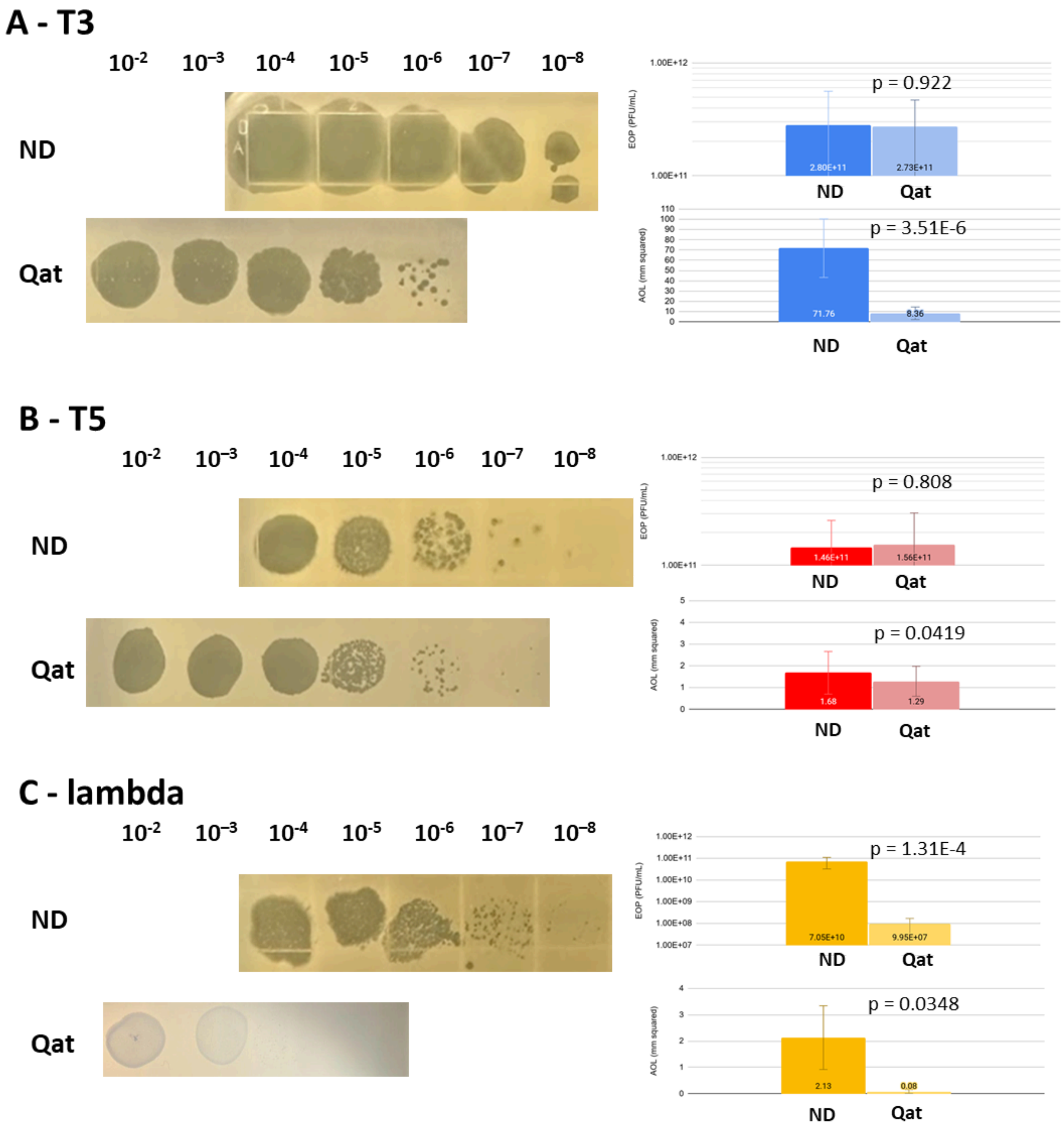


**Raw Data 2. Serial dilution plaque formation assay**

Phages T3 (**A**), T5 (**B**), and lambda (**C**) were assayed against *E. coli* MG1655 carrying either an empty vector (no defense, ND) or the Qat defense system. For each phage, a representative dilution spot assay is shown alongside two graphs quantifying the efficiency of plating (EOP) and the area of lysis (AOL). EOP data represent the mean of four replicates, and AOL data represent the mean of three plaques measured

per replicate. Statistical significance was determined using a Student's t-test comparing the Qat strain to the ND control, with *P*-values indicated on the graphs.
